# Supplementary material for: Polyketide synthase gene clusters in the ericoid mycorrhizal fungus Oidiodendron maius: genome-wide prediction, expression profiling, and functional characterization of knockout mutants
Source: Front Plant Sci. 2026 Sep 16;17:1924430. doi: 10.3389/fpls.2026.1924430 (PMC13623903; doi:10.3389/fpls.2026.1924430)
Supplement: Supplementary file 1 [file SupplementaryFile1.zip › Supplementary Materials/Supplementary Folder 1.zip!/Oidma1_AssemblyScaffolds_Repeatmasked/knownclusterblast/cluster34/jgi.p_Oidma1_58393_mibig_hits.html]

| MIBiG Protein | Description | MIBiG Cluster | MiBiG Product | % ID | % Coverage | BLAST Score | E-value |
| --- | --- | --- | --- | --- | --- | --- | --- |
| AAN74819.2 | Fum16p | BGC0000062 | Fumonisin\_biosynthetic\_gene\_cluster | 48 | 98.4126984127 | 643 | 2.3e-184 |
| ACB12561.1 | Fum16 | BGC0000063 | Fumonisin\_biosynthetic\_gene\_cluster | 47 | 97.6911976912 | 630 | 2.6e-180 |
| AFO59871.1 | ChxJ | BGC0000175 | Cycloheximide\_/\_actiphenol\_biosynthetic\_gene\_cluster | 29 | 67.8210678211 | 159 | 1.7e-38 |
| AEZ64574.1 | fatty-acid-CoA\_ligase | BGC0001065 | Herboxidiene\_biosynthetic\_gene\_cluster | 24 | 85.2813852814 | 152 | 1.6e-36 |
| AHJ31217.1 | Long-chain-fatty-acid--CoA\_ligase | BGC0000430 | Spumigin\_biosynthetic\_gene\_cluster | 24 | 68.5425685426 | 87 | 4.8e-17 |
| AIL50189.1 | putative\_5-aminolevulinate\_CoA-ligase | BGC0000213 | Colabomycin\_biosynthetic\_gene\_cluster | 35 | 28.2828282828 | 83 | 9.1e-16 |
| AFV30247.1 | polyketide\_synthase | BGC0000075 | Hygrocin\_biosynthetic\_gene\_cluster | 35 | 20.3463203463 | 83 | 9.1e-16 |
| ABJ90146.1 | MoeA4 | BGC0000805 | Moenomycin\_biosynthetic\_gene\_cluster | 36 | 23.5209235209 | 82 | 2.6e-15 |
| ADC79613.1 | BafX | BGC0000028 | Bafilomycin\_biosynthetic\_gene\_cluster | 37 | 23.5209235209 | 81 | 5.9e-15 |
| AKD43499.1 | Acyl-CoA\_ligase | BGC0001409 | Dutomycin\_biosynthetic\_gene\_cluster | 31 | 31.1688311688 | 76 | 1.1e-13 |
| ACN64850.1 | PokL | BGC0001061 | Polyketomycin\_biosynthetic\_gene\_cluster | 33 | 23.088023088 | 76 | 1.5e-13 |
| AFU65902.1 | DacH | BGC0000216 | Dactylocycline\_biosynthetic\_gene\_cluster | 31 | 24.531024531 | 75 | 2.5e-13 |
| AGL76720.1 | fatty\_acid\_CoA\_ligase | BGC0001137 | Marinacarboline\_biosynthetic\_gene\_cluster | 26 | 47.619047619 | 73 | 9.4e-13 |
| AAZ03552.1 | McnC | BGC0000332 | Cyanopeptolin\_biosynthetic\_gene\_cluster | 25 | 56.5656565657 | 69 | 1.8e-11 |
| BAH43870.1 | putative\_linear\_pentadecapeptide\_gramicidin\_synthetase\_LgrB | BGC0000367 | Gramicidin\_biosynthetic\_gene\_cluster | 23 | 75.1803751804 | 68 | 4e-11 |
| BAH22764.1 | nonribosomal\_peptide\_synthetase | BGC0001018 | Micropeptin\_biosynthetic\_gene\_cluster | 24 | 54.5454545455 | 68 | 4e-11 |
| BAF50727.1 | hybrid\_polyketide\_synthase-non\_ribosomal\_peptide\_synthetase | BGC0001116 | Virginiamycin\_biosynthetic\_gene\_cluster | 24 | 57.2871572872 | 66 | 2e-10 |
| ADN26257.1 | ATP-dependent\_adenylase | BGC0000951 | Pacidamycin\_biosynthetic\_gene\_cluster | 24 | 51.3708513709 | 65 | 3.3e-10 |
| ADI58645.1 | AsuD1 | BGC0000187 | Asukamycin\_biosynthetic\_gene\_cluster | 30 | 23.8095238095 | 64 | 4.4e-10 |
| CAE02600.1 | putative\_acyl-coA\_ligase | BGC0000024 | Aureothin\_biosynthetic\_gene\_cluster | 28 | 25.2525252525 | 64 | 5.7e-10 |
| ADY16697.1 | TqaA | BGC0001142 | Tryptoquialanine\_biosynthetic\_gene\_cluster | 30 | 26.6955266955 | 64 | 5.7e-10 |
| AIL50191.1 | putative\_amide\_synthetase | BGC0000213 | Colabomycin\_biosynthetic\_gene\_cluster | 32 | 24.2424242424 | 64 | 7.5e-10 |
| EAW16180.1 | nonribosomal\_peptide\_synthase,\_putative | BGC0000293 | Acetylaszonalenin\_biosynthetic\_gene\_cluster | 28 | 24.2424242424 | 62 | 2.2e-09 |
| ACZ66258.1 | APS1 | BGC0000304 | Apicidin\_biosynthetic\_gene\_cluster | 29 | 23.5209235209 | 59 | 1.8e-08 |
| AIG79243.1 | Hypothetical\_protein | BGC0000419 | Ristomycin\_A\_biosynthetic\_gene\_cluster | 24 | 53.5353535354 | 58 | 3.1e-08 |
| ABK32262.1 | AmbG | BGC0000014 | Ambruticin\_biosynthetic\_gene\_cluster | 24 | 51.9480519481 | 56 | 1.2e-07 |
| CBJ90082.1 | Non\_Ribosomal\_peptide\_synthetase\_(-succinylbenzoate--CoA\_ligase) | BGC0001132 | Xenotetrapeptide\_biosynthetic\_gene\_cluster | 23 | 54.2568542569 | 56 | 1.6e-07 |
| ABL74936.1 | TlmX | BGC0001048 | Tallysomycin\_biosynthetic\_gene\_cluster | 26 | 50.2164502165 | 56 | 1.6e-07 |
| AFJ23826.1 | WLIP\_synthetase\_C | BGC0000462\_c2 | WLIP\_biosynthetic\_gene\_cluster | 29 | 25.8297258297 | 55 | 3.5e-07 |
| CCC55922.1 | putative\_non-ribosomal\_peptide\_synthetase | BGC0000973 | Collismycin\_A\_biosynthetic\_gene\_cluster | 27 | 31.4574314574 | 51 | 3.8e-06 |
| AAN32979.1 | BarE | BGC0000962 | Barbamide\_biosynthetic\_gene\_cluster | 25 | 25.8297258297 | 51 | 5e-06 |
